# Reproductive Organ and Vascular Specific Promoter of the Rice Plasma Membrane Ca^2+^ATPase Mediates Environmental Stress Responses in Plants

**DOI:** 10.1371/journal.pone.0057803

**Published:** 2013-03-01

**Authors:** Kazi Md. Kamrul Huda, Mst. Sufara Akhter Banu, Krishna Mohan Pathi, Narendra Tuteja

**Affiliations:** Plant Molecular Biology Group, International Centre for Genetic Engineering and Biotechnology, Aruna Asaf Ali Marg, New Delhi, India; University of South Florida College of Medicine, United States of America

## Abstract

**Background:**

Plasma membrane Ca^2+^ATPase is a transport protein in the plasma membrane of cells and helps in removal of calcium (Ca^2+^) from the cell, hence regulating Ca^2+^ level within cells. Though plant Ca^2+^ATPases have been shown to be involved in plant stress responses but their promoter regions have not been well studied.

**Results:**

The 1478 bp promoter sequence of rice plasma membrane Ca^2+^ATPase contains cis-acting elements responsive to stresses and plant hormones. To identify the functional region, serial deletions of the promoter were fused with the GUS sequence and four constructs were obtained. These were differentially activated under NaCl, PEG cold, methyl viologen, abscisic acid and methyl jasmonate treatments. We demonstrated that the rice plasma membrane Ca^2+^ATPase promoter is responsible for vascular-specific and multiple stress-inducible gene expression. Only full-length promoter showed specific GUS expression under stress conditions in floral parts. High GUS activity was observed in roots with all the promoter constructs. The −1478 to −886 bp flanking region responded well upon treatment with salt and drought. Only the full-length promoter presented cold-induced GUS expression in leaves, while in shoots slight expression was observed for −1210 and −886 bp flanking region. The −1210 bp deletion significantly responded to exogenous methyl viologen and abscisic acid induction. The −1210 and −886 bp flanking region resulted in increased GUS activity in leaves under methyl jasmonate treatments, whereas in shoots the −886 bp and −519 bp deletion gave higher expression. Salicylic acid failed to induce GUS activities in leaves for all the constructs.

**Conclusions:**

The rice plasma membrane Ca^2+^ATPase promoter is a reproductive organ-specific as well as vascular-specific. This promoter contains drought, salt, cold, methyl viologen, abscisic acid and methyl jasmonate related cis-elements, which regulated gene expression. Overall, the tissue-specificity and inducible nature of this promoter could grant wide applicability in plant biotechnology.

## Introduction

Abiotic stress (drought, salt, cold, high and low temperature, water deficiency or excess) is the most harmful factor for growth and productivity in crops worldwide. These problems increase consistently due to climate change. Many researchers have obtained transgenic plants with improved stress tolerance by overexpressing genes with stress-protecting functions [1]. However, in some cases, the transgenic plants display undesirable side effects, such as low yield [2], delayed growth [3], and dwarfism [4]. These phenotypes might be due to transgene ectopic expression. To avoid such a problem, tissue-specific or stress-inducible promoters and their upstream regulatory elements need to be studied extensively. During abiotic stress a signal transduction pathway starts with signal perception, followed by generation of second messenger like Ca^2+^ ion. Second messengers are involved in phosphorylation cascade which ultimately leads to activation of transcription factors controlling sets of stress regulated genes.

As a second messenger of paramount significance, Ca^2+^ is required in almost all the stages of plant growth and development, playing a fundamental role in regulating polar growth and participating in plant adaptation to various stress factors [5]. Under stress conditions, Ca^2+^ plays crucial roles in plant membrane stability, cell wall stabilization, and cell integrity [6]. It also acts as sensor of multiple and variable environmental signals, resulting in widely mediated stimulus-response coupling by modulation of cytosolic free Ca^2+^ [7]–[10], which in turn is modulated by calcium/calmodulin mediated proteins (Ca^2+^/CaM). It has been well evidenced that various environmental conditions and hormone signal molecules as light stress, low temperatures, salt, alkali, gibberellins, or abscisic acid (ABA) can trigger alteration of cytosolic Ca^2+^ concentrations, leading to different plant adaptation responses [11], [12]. Plant Ca^2+^ATPases are members of p-type ATPase superfamily involved in the restoration and maintenance of ion homeostasis by pumping calcium ions out of the cytosol in all eukaryotic cells. Plant p-type Ca^2+^ATPases have been divided into two groups, type IIA and IIB. The latter contains an N-terminal autoinhibitory domain that binds to calmodulin and activates the Ca^2+^pump [13].

The central role in calcium signalling seems to be played by Ca^2+^ATPases and the expression levels of different plant Ca^2+^ATPase genes (*Arabidopsis Ca^2+^ATPase 4, Soybean Ca^2+^ATPase 1, Lycopersicon Ca^2+^ATPase 1 and Physcomitrella Ca^2+^ATPase 1*) were shown to be up-regulated under salinity stress, [14]–[17]. In *Arabidopsis* ACAs, type IIB Ca^2+^ATPases, may be involved in cytosolic Ca^2+^ signal shaping in response to several stresses. For example, *AtACA8* was found to be up-regulated, whereas *AtACA10* was down-regulated in response to cold stress [18]. Under low temperature (2°C) stress, the activity and stability of Ca^2+^ATPase plays key functions in the development of cold resistance in winter wheat [19]. Earlier, it is suggested that CR-4, a cold-resistant agent (introduced by Plant Research Institute of Chinese Academy of Sciences), plays a momentous role in stabilizing plasma membrane Ca^2+^ATPase under low temperature stress, indicating that the Ca^2+^ATPase activity was mainly localized at the plasma membrane in wheat seedling cells growing at normal temperatures [20]. Type IIB Ca^2+^ATPases present Ca^2+^/CaM binding regions, contributing to ABA-induced drought signal transferring under PEG stress, since ABA synthesis was related with cytoplasmic Ca^2+^ concentrations [21]. It was reported that ABA triggers an increase in cytosolic Ca^2+^ in guard cells, including Ca^2+^ influx across the plasma membrane [22]. In *Arabidopsis*, the expression of *ACA8* and *ACA9* genes might be stimulated by ABA, suggesting for an indirect role of plant Ca^2+^ATPases in stress signalling [23]. Previously it was shown that Ca^2+^/CaM messenger system was involved in controlling stress resistance in rice seedling, blocking messenger transduction, drought resistance, salt resistance and decreasing chilling resistance [24]. It was also indicated that Ca^2+^ treatment increased protection against membrane lipid peroxidation and membrane stability and therefore resulted in the increase of drought resistance in rice seedlings [25]. In wheat, Ca^2+^ appeared to reduce the devastating effects of stress by elevating the content of proline and glycine betaine, thus improving the water status and growth and minimizing the injury to membranes [26]. Above-mentioned results showed that Ca^2+^ plays important roles in plant responses to drought resistance. Previously, Romani [27] showed that low concentration of Eosin Yellow (type IIB Ca^2+^ATPase inhibitor) prevented both the increase in Ca^2+^ efflux and the transient reactive oxygen species (ROS) accumulation in *Egeria densa* in response to ABA treatment. This result was explained by assuming an important role of PMCa^2+^ATPase in switching off the signal triggering ROS production. Another report from the same group implicated PMCa^2+^ATPase activation in plant adaptation to osmotic stress [28]. Plant Ca^2+^ATPase not only regulates plant development and abiotic stresses, but also protects plant from pathogens by activating salicylic acid (SA)-mediated programmed cell death (PCD) pathways [29]. These results provide the evidence for the importance of Ca^2+^ATPases in shaping cytosolic Ca^2+^ signatures under abiotic and biotic stresses.

Considering the key role played by Ca^2+^ATPases in the plant ability to tolerate abiotic stress, it is desirable and feasible to exploit stress-inducible promoters to drive the expression of relevant transgenes. In this study, we isolated and analyzed a stress inducible promoter OsPMCa^2+^ATPase from rice and investigated it with regards to tissue specific expression pattern and relative expression activities, using transgenic analysis in tobacco under different stresses. We also identified the shortest promoter region by making random deletion and found promoter regions sufficient for tissue specific expression and stress induced expression activity. This promoter drives high levels of transgene expression under abiotic stress conditions and will be useful for the development of stress tolerant transgenic plants.

## Materials and Methods

### Analysis of Promoter Sequences

DNA sequences were analysed by using DNAMAN software, while PLANT CARE (http://bioinformatics.psb.ugent.be/webtools/plantcare/html/, 29) and PLACE (http://www.dna.affrc.go.jp/PLACE/, 30) were used to determine the cis-acting regulatory elements and to analyze the OsPMCa^2+^ATPase promoter sequences (http://rice.plantbiology.msu.edu).

### Amplification of OsPMCa^2+^ATPase and Construction of Chimeric Promoter

Genomic DNA was extracted from leaves of *Oryza sativa* (Var. IR 64) by CTAB method and used as template for PCR amplification of OsPMCa^2+^ATPase promoter. Sequences of DNA adaptors and primers used for promoter amplification are provided in Table S1. Deletions were made at the 5′-upstream end, based on the distribution of structural and expressional elements of the known OsPMCa^2+^ATPase promoter sequence. To construct the various promoter deletions of OsPMCa^2+^ATPase promoter with GUS fusion products, a PCR series was carried out with four primer pairs, F/RS, D1/RS, D2/RS and D3/RS (Table S1), respectively. The amplified fragments were then cloned into pGEMTeasy vector. Four different promoter deletions were released by BamHI and HindIII digestion and then cloned in pCAMBIA-1391Z (promoter less vector) in the same restriction site. Four expression vectors containing various promoter deletions of OsPMCa^2+^ATPase promoter were individually obtained and designated full-length (F), D1, D2, and D3. In addition, the CaMV35S promoter was used as positive control and wild-type tobacco as negative control, in order to determine OsPMCa^2+^ATPase promoter activity.

### Tobacco Transformation and PCR Analysis

Tobacco (*Nicotiana tabacum* cv. Xanthi) leaf discs were transformed using a standard procedure as described earlier [30] with *Agrobacterium tumefaciens* (LBA4404) containing promoter-GUS (β-glucuronidase) fusion constructs in pCAMBIA-1391Z. Primary transgenic explants were grown in tissue culture chamber at 26°C under a 16 h light/8 h dark cycle. The transgenic plants were screened for integration of the intact promoter-GUS chimeric gene into the genome by PCR. PCR products were analysed on 1% (w/v) agarose gel. Total genome DNA was isolated from leaves of hygromycin resistant tobacco plants using the CTAB method. PCR analysis was carried out using F/RS, D1/RS, D2/RS and D3/RS primers, hygromycin specific primers and GUS specific primer pair (Table S1).

### Abiotic Stresses and Hormone-induced Treatments in Transgenic Tobacco

Transgenic tobacco plants were grown in greenhouse at 24–26°C. Two months old plants were used for induction of stress treatments. Salt, drought, cold, abcisic acid ABA, SA, methyl jasmonate (MeJA) and methyl viologen (MV) treatments were chosen. Flowers, stems, leaves and roots of transgenic tobacco were cut into pieces and subjected to soaking in Petri dishes filled with 200 mM NaCl, 20% PEG, 100 µM ABA, 2 mM SA, 200 µM MeJA and 100 µM MV solution or sterilized water, for 24 h at room temperature. For cold stress, tissues were incubated at 4°C for 48 h. Tissues in absence of stress were used as control. All the above treatments were carried out under a growth regime of 16 h light/8 h dark at 20±1°C unless otherwise mentioned.

### Histochemical GUS Staining

Leaves, shoots, roots and flowers were vacuum infiltrated for 1 h in the GUS reaction mixture containing 2 mM 5-bromo-4-chloro-3-indolyl- β-D-glucuronide (X-Gluc) and 100 mM sodium phosphate buffer as described by Jefferson [31], and incubated at 37°C overnight. The reaction was stopped by adding 75% ethanol, and the pigments and chlorophylls were removed by repeated ethanol washing. In addition, roots, leaf sheaths and stalks were sectioned manually with a razorblade and the sections stained with X-Gluc as described by Jefferson [31]. The images of blue-coloured whole plants were pictured by a Sony Cyber-shot camera. The GUS-positive plant tissues were examined with NIKON AZ 100 microscope at a low magnification and NIKON digital SIGHT DS-Ri 1camera and images was analyzed by NIS-Elements A.R software. GUS-stained tissues and plants in the present paper represent the typical results of at least three independent transgenic lines for each construct.

### Protein Extraction and GUS Fluorometric Analysis

The behavior of OsPMCa^2+^ATPase promoter induced by various stresses was studied using transgenic tobacco seedlings. Fluorometric analysis of GUS activity was performed using 4-methylumbelliferyl-b-glucuronide (4-MUG). The extracted proteins were mixed with GUS assay buffer (2 mM 4-MUG, 50 mM sodium phosphate buffer pH 7.0, 10 mM β-mercaptoethanol, 10 mM Na_2_EDTA, 0.1% sodium lauroyl sarcosine, and 0.1% Triton X-100). The addition of the stop buffer (0.2 M Na_2_CO_3_) halted the reaction. Next, 4-MUG was hydrolyzed by GUS to produce 4-methylumbelliferone fluorochrome (4-MU). GUS activity was determined in triplicate with a microplate spectrofluorometer (1420 Multilabel counter, Perkin Elmer, Finland). The excitation wavelength was 365 nm and the emission wavelength 455 nm. Protein concentration was determined using Bradford assay, according to the manufacturer’s instructions.

## Results

### Isolation of the OsPMCa^2+^ATPase Promoter from Rice and Cis-elements Analysis

**Figure 1:**
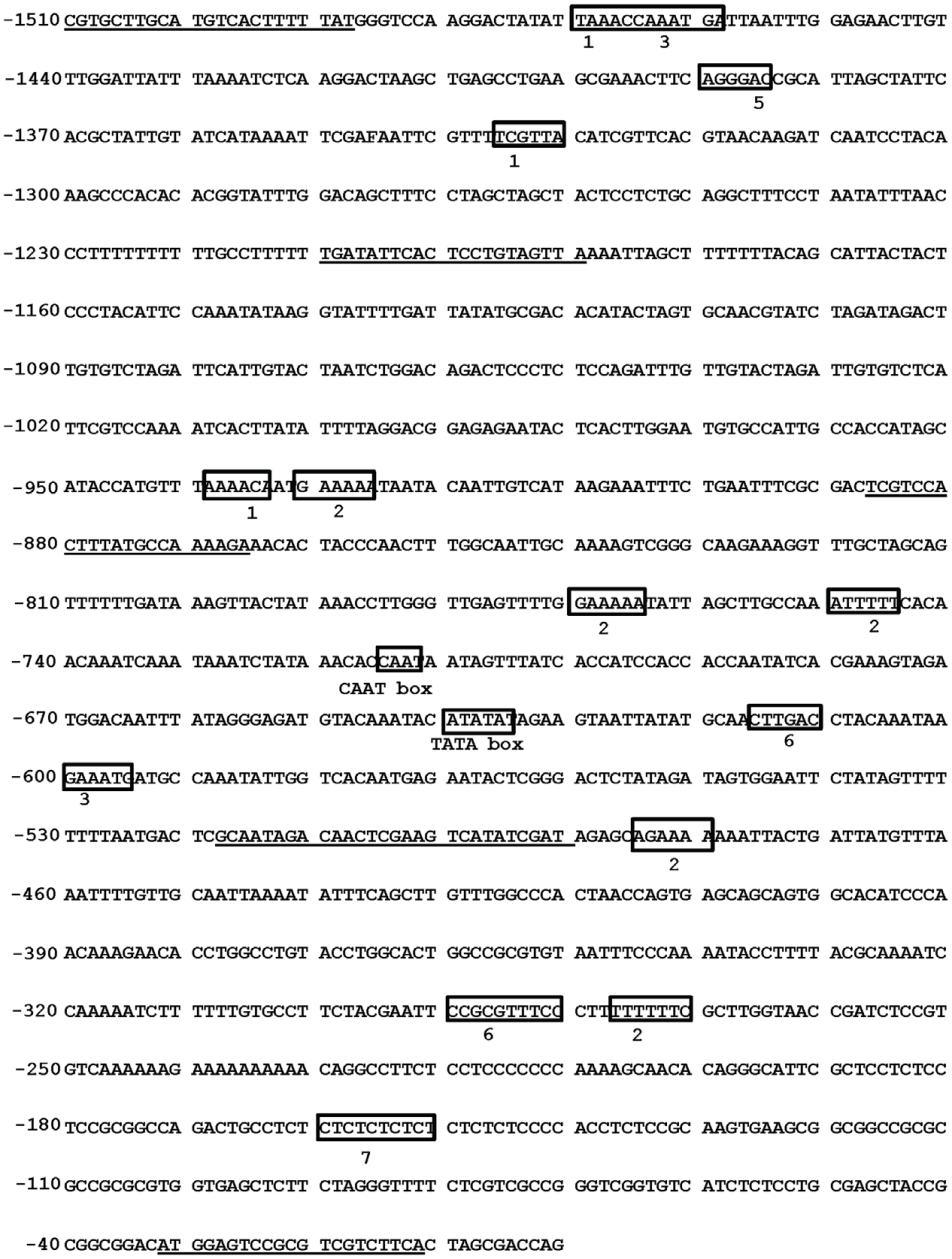
OsPMCa^2+^ATPase promoter sequence. Underlined sequence: primers designed for deletion analysis 1. MYB recognition site; 2. GT1GMSCAM4: pathogen- and salt-responsive element; 3. MYC recognition site; 4. ABRERATCAL: Ca^2+^responsive element; 5: DRE recognition site; 6: WBOXATNPR1: SA-responsive element; 7: CTRMCAMV35S: enhancer.

Based on the annotation of rice genome, the −1478 bp fragment of OsPMCa^2+^ATPase promoter was isolated from *Oryza sativa* genomic DNA using the OsPMCa^2+^ATPase specific primer sets (Table S1). The −1478 bp PCR product was cloned into vector pCAMBIA 1391Z, a promoter less vector, in order to use it for tobacco plant transformation. Successful insertion was confirmed by sequencing. In order to identify the cis-acting elements involved in the response to various stress condition we analysed the activity of cis element using PLACE and PLANTCARE databases (Figure 1). The 1478 bp promoter region upstream of the OsPMCa^2+^ATPase start codon contains various putative cis-elements and we analyzed only cis-elements in boxes known to be related to abiotic stress and hormone signalling (Figure 1). Predicted cis-elements present in OsPMCa^2+^ATPase promoter using database analysis was shown to harbour multiple stress cis-acting elements (Table 1). Six homologue sequences of the pathogenesis- and salt-related cis-acting element GT1GMSCAM4 (GAAAAA) were evidenced. One homologue sequence of GAREAT (TAACAAR), an abundant sequence upstream of GA-induced genes in *Arabidopsis* [32], was present in rice. Two homologue sequences of WBOXATNPR1 (TTGAC) were also detected. Two homologue sequences of WBOXHVISO1 (TGACT), which mainly participates in sugar signal transduction [33], were found. In addition, light-responsive elements such as I-BOX (GATTA), GT1 (GRWAAW) and GATABOX (GATA) were present. Only two homologue sequences of ABRERATCAL (MACGYGB) element, a calcium responsive cis-element were discovered. ABRE is a cis element that involved in transcriptional activation in response to cytosolic Ca^2+^ transients. A recent transcriptomic analysis revealed that out of 230 calcium-responsive genes, 162 upregulated genes is contained ABRE cis element of their upstream regions [34]. Various elements responsible for ABA, drought, cold, dehydration and low temperature were individuated. The analysis showed that most elements present in OsPMCa^2+^ATPase promoter were mainly environmental or hormone-responsive motifs. In conclusion, it can be predicted that OsPMCa^2+^ATPase promoter could be an inducible promoter, regulated by multiple abiotic factors and hormones.

**Table 1:** Predictions of cis-elements present in OsPMCa^2+^ATPase promoter using PLANT CARE database analysis.

Element	Element core sequence	Element Number	Function
ABRERATCAL	MACGYGB	2	Response to calcium ion
CGCGBOXAT	VCGCGB	16	Involved in multiple signal transduction
CTRMCAMV35S	TCTCTCTCT	5	plays as enhancer
CURECORECR	GTAC	8	copper and oxygen signals
CBFHV	RYCGAC	1	Response to low temperature
CCAATBOX1	CCAAT	3	Element for heat sock
DPBFCOREDCDC3	ACACNNG	4	Response to ABA signals
GT1GMSCAM4	GAAAAA	6	Pathogenesis and salt related element
MYB	WAACCA/CTAACCA/CNGTTR	4	response to drought and ABA signals
MYC	CANNTG	12	response to drought, ABA and cold signals
DRE	ACCGCA/RCCGAC	1	Drought, ABA high salt and cold responsive element
GAREAT	TAACAAR	1	Response to GA signals
WBOXATNPR1	TTGAC	2	Element for biotic and environmental stress
WBOXHVISO1	TGACT	2	Response to sugar signals
WBOXNTERF3	TGACY	4	Response to wounding
GATABOX	GATA	10	Response to light
GT1CONSENSUS	GRWAAW	10	Response to light
IBOXCORE	GATAA	3	Response to light
INRNTPSADB	YTCANTYY	1	Response to light
WBOXATNPR1	TTGAC	2	Response to SA signal
ABRELATERD1	ACGTG	1	Response to dehydration stress
ARFAT	TGTCTC	1	Response to auxin signalling
LTRECOREATCOR15	CCGAC	2	Response to low temperature, cold, drought, ABA
TAAAGSTKST1	TAAAG	2	regulate guard cell-specific gene expression

### Transgenic Tobacco Plants Harboring OsPMCa^2+^ATPase Promoter were Generated

**Figure 2:**
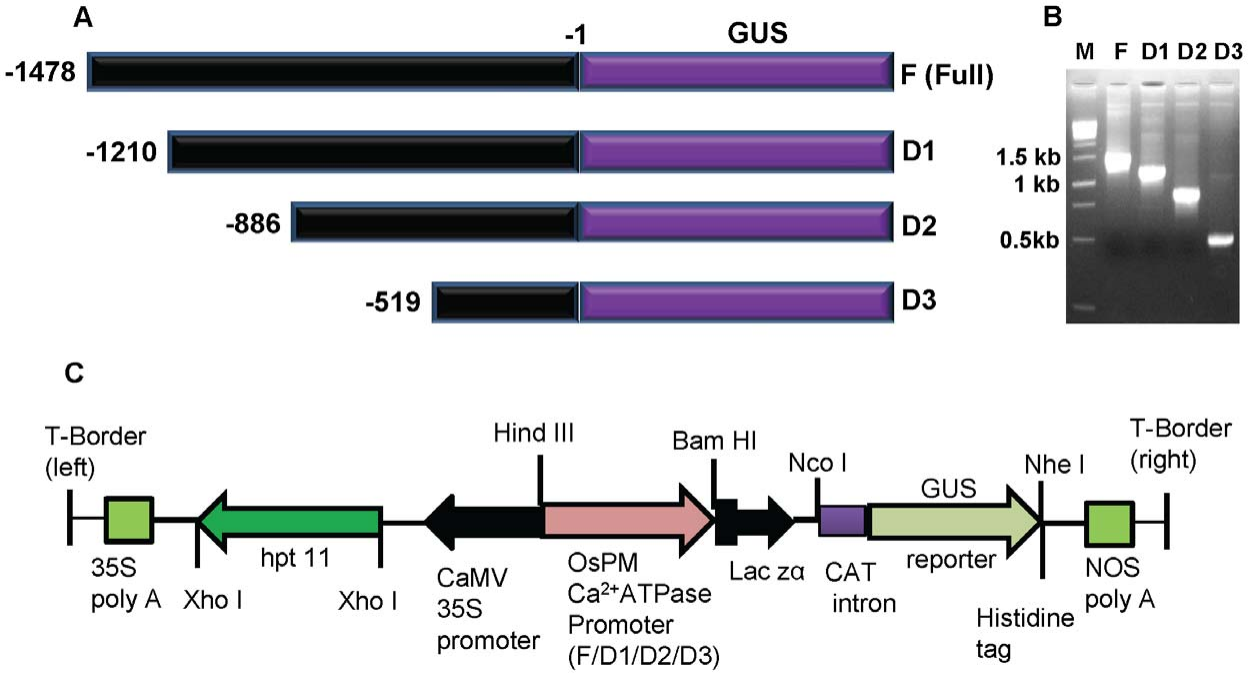
OsPMCa^2+^ATPase promoter deletion analysis and schematic representation of different constructs. **A)** OsPMCa^2+^ATPase promoter deletion analysis**:** The first base before ATG represents −1; F, full-length PM Ca^2+^ATPase promoter; D1, F 5′-end deletion of 300 bp; D2, F 5′-end deletion of 624 bp; D3, F 5′-end deletion of 991 bp. **B)** Amplification OsPMCa^2+^ATPase promoter with their different deletions. **C)** Schematic representation Ca^2+^ATPase promoter in pCAMBIA 1391Z (a promoter less vector) for raising transgenic tobacco.

To identify the regions of OsPMCa^2+^ATPase promoter which are active in the response to different abiotic stress, serial deletions were created (Figure 2A). Deletions, beginning at positions −1210, −886, −519 along with the intact promoter fragment extending to position −1478 (Figure 2B) were fused with GUS sequence and separately transferred into tobacco by *Agrobacterium* mediated leaf-disk transformation. The promoter region (full-length and their deletions, D1, D2 and D3) was cloned at HindIII and BamHI sites of promoter less vector, pCAMBIA1391Z (Figure 2C). Transgenic tobacco lines containing full-length promoter, D1, D2, D3 deletions and CaMV35S-GUS were obtained. In case of full-length promoter six positive lines were obtained while D1, D2 and D3 deletions gave seven, eight and five respectively (Figure S1). Three independent hygromycin resistant PCR-positive transgenic tobacco plants were chosen from each group for further analysis.

### Fluorometric Quantification and Expression of GUS Activity in Transgenic Tobacco

**Figure 3:**
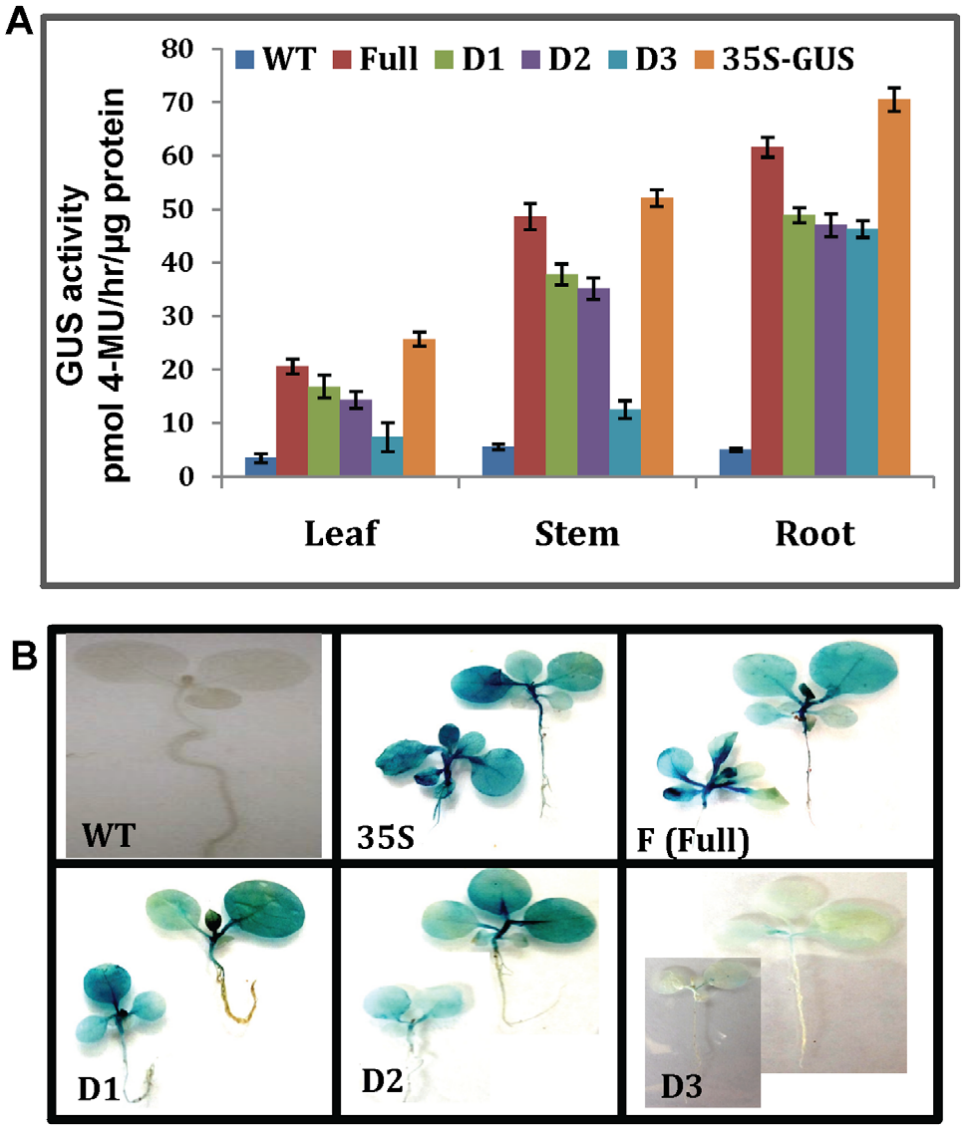
Fluorometric quantification and histochemical analysis of GUS activity. **A)** GUS enzyme activity among different transgenic groups in leaves stems and roots. The GUS activity is expressed in pmol–4 MU/hr/µg protein, and the graphic drawn the average rate of GUS activity per collection of transgenics per construct. The quantification of GUS activity for each promoter construct was replicated three times. Error bars on the graph represent SE within the three replicates. **B)** GUS expression activity was detected by vacuuming seedlings in X-Gluc solution overnight using two weeks old transgenic tobacco plantlets, directed by OsPMCa^2+^ATPase promoter construct F, D1, D2 and D3. For positive and negative controls plants transformed with the 35S promoter and wild type (WT) plants were used separately.

In order to evaluate the GUS activity in the transgenic tobacco lines harboring full-length promoter along with promoter deletions, a fluorimetric assay was used. As a first analysis, the expression of OsPMCa^2+^ATPase promoter and deletions was assessed in transgenic tobacco leaves, stems and roots under normal conditions. Results are showed in Figure. 3A. In general, the GUS-specific signal was very high in roots (∼3 fold increase) and stems (∼2 fold increase) as compare to leaves. As expected, the full-length promoter segment presented the highest GUS activity when compared with deletions. However, it expression was lower than the positive control CaMV35S promoter in all tested tissues. Results of the GUS assay showed that removing the −519 bp significantly affected quantitative behaviour; GUS activity decreased sharply with −519 bp deletion (Figure 3A). This might be due to the −519 bp deletion contain an enhancer-like cis-element. The fluorescence quantification data correlated with the histochemical staining results. Together with the GUS staining results, we demonstrated that the deletion sequence were required for both tissue specificity and quantitative behaviour.

To identify the expression profiles of transgenic plants driven by the four OsPMCa^2+^ATPase promoter deletions, tobacco plants were subjected to histochemical staining. Results are shown in Figure 3B. GUS expression was detected in one week old etiolated seedlings of full-length promoter and deletion transgenics. The CaMV35S promoter, used as positive control, was expressed in all tobacco tissues, while no GUS activity was detected in wild-type tobacco plants. GUS expression activity of full-length promoter was the strongest among the four OsPMCa^2+^ATPase promoter constructs examined. −1210 bp and −886 bp deletions had similar expression patterns. No GUS activity was evidenced in the case of the longest deletion (−519 bp) (Figure 3B). The results suggest that OsPMCa^2+^ATPase promoter deletions −1210 bp and −886 bp are sufficient to drive gene expression.

### Drought, Salt and Cold Stress Induces GUS Activity of Promoter(s) in Tobacco Plants

**Figure 4:**
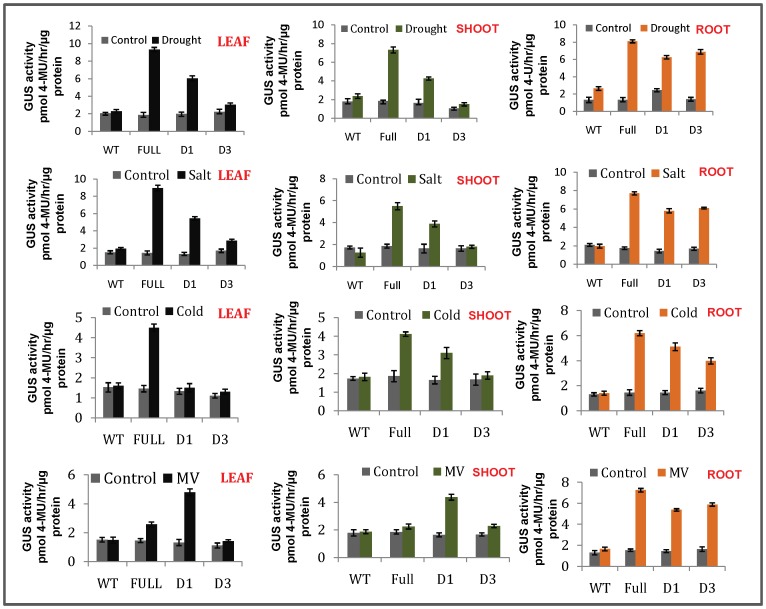
Quantification of GUS relative activity of OsPMCa^2+^ATPase promoter deletions under drought, salt cold and MV induced stress in leaves shoots and roots of tobacco. The transgenic tobacco plants driven by promoter-GUS fusion constructs full-length (−1478 bp), D1 (−1210 bp) and D3 (−519 bp) were chosen for quantification assays. The deletion construct D2 was also used for the analysis but the data of D2 was similar to the D1, therefore the D2 data is omitted in this figure because of the space constraint. Wild type (WT) tobacco plants with same treatment were used as the control. Data of three independent transgenic lines were measured, and each experiment was replicated three times. Error bars on the graphic represent SE with three replicates.

Since it was shown that abiotic stresses can regulate OsPMCa^2+^ATPase gene expression, its promoter activity was studied in tobacco transgenic plants submitted to drought, salt and cold treatments. The presence of abiotic-stress-responsive elements inside the promoter sequence encouraged the research. In consequence, GUS activity was measured through fluorometric assay. Distinct GUS expression was evidenced for each of the four (full-length, −1478 bp; D1, −1210 bp; D3, −519 bp and wild type) OsPMCa^2+^ATPase promoter segments (Figure 4). The results of deletion construct D2 were similar to the D1, therefore the data of D2 is omitted in the figures 4 because of the space constraint. In roots, high GUS expression was observed in full-length and in all the deletions (∼2–3.5 fold increase) after treatment with stress. Reckonable increase of GUS activity in leaves (∼4.5 fold) and shoots (∼3 fold) was evidenced for full-length promoter, while −1210 bp showed ∼2.8 fold increase in leaves and ∼2 fold increase in shoots compared with control upon treatment with drought, while no activity was observed in −519 bp deletion. This may be due to presence of some inhibitory elements upstream of −519 bp segment that affect its expression. Similar results were presented in case of salt-induced stress. In leaves and shoots cold shock induced ∼2.8 fold increase GUS expression only for full-length promoter while in −1210 bp and −519 bp no expression was observed in leaves. Under same conditions, a slight induction was present in −1210 bp (∼2.8 fold increase *GUS* activity) deletions in shoots (Figure 4). Thus, we conclude that the full OsPMCa^2+^ATPase promoter positively responded to drought, salt and cold. Since deletion −1210 bp and −519 bp did not respond to cold whereas the full-length promoter did, it might result from the absence of cold-related cis-acting elements in these segments. We suggest that cold-related cis-elements may be required in response to cold in leaves outside the region contained in the −1210 bp deletion.

### Oxidative Stress-induced GUS Activity in Tobacco Transgenic Plants

To provide further evidence for the idea that the OsPMCa^2+^ATPase is responsive to oxidative stress, tobacco plants were treated with MV, compound known to alter the cellular ROS concentration. To evaluate the effect of MV on the expression of GUS, leaf, shoot and root samples of all promoter constructs were treated with MV for 24 hr. Results are presented in Figure 4. Approx. 3.5–5 fold increase *GUS* expression was observed by MV induction in root when compared to control. In leaves and shoots, out of the three construct only −1210 bp promoter deletion significantly responded to exogenous MV induction showing approx. 3 fold increase *GUS* expression (Figure 4).

### Hormone-induced GUS Activity

**Figure 5:**
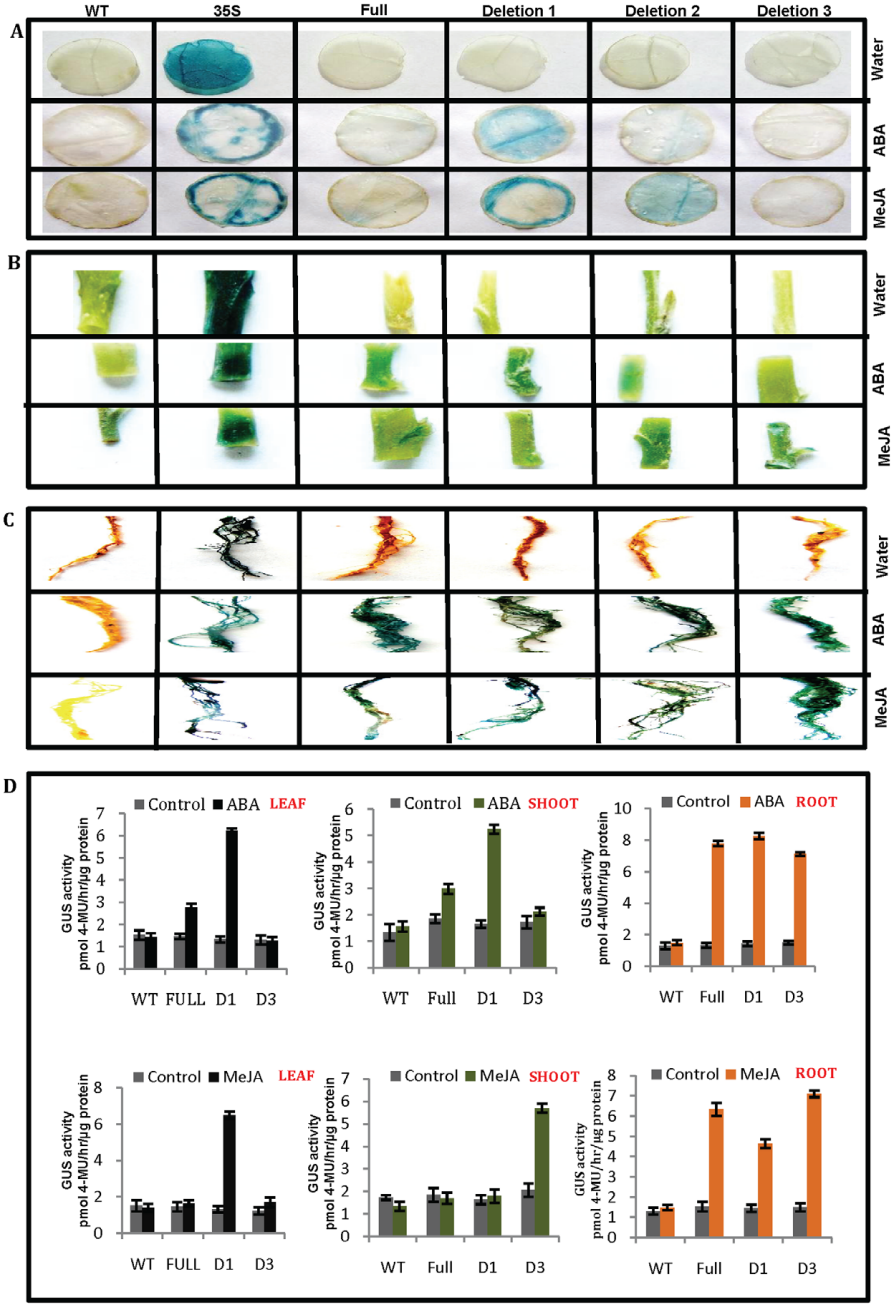
Gus expression analysis and estimation of relative *GUS* activity of various OsPMCa^2+^ATPase promoter deletions under ABA and methyl jasmonate (MeJA)-induced stress. A) Gus activity in leaves, **B)** Gus activity in shoots and **C)** Gus activity in roots. Gus was detected in X-Gluc solution using mature leaf disc, shoots cutting and roots from transgenic tobacco plant, directed by promoter constructs full-length (−1478 bp), D1 (−1210 bp), D2 (−886 bp) and D3 (−519 bp). For positive and negative controls 35S and WT were used. **D)** The transgenic tobacco plants driven by promoter-GUS fusion constructs full-length, D1 and D3 were chosen for quantification assays. The deletion construct D2 was also used for the analysis but the data of D2 was similar to the D1, therefore the D2 data is omitted in this figure because of the space constraint. Wild type (WT) tobacco plants with same treatment were used as control. Leaf, stem and root samples from transgenic lines were used. Data were measured in three independent transgenic lines, and each experiment was replicated three times. Error bars on the graphic represent SE within the three replicates.

Leaves, shoot and roots were separated from transgenic plants and hormones were applied to examine GUS expression for each of the four OsPMCa^2+^ATPase promoter segments. *GUS* expression responded differently to ABA, SA and MeJA-induced treatments are shown in Figure 5. In leaves, among all the promoter segments, only −519 bp deletion significantly responded to exogenous ABA induction whereas full-length (−1478 bp) promoter and −886 bp deletion showed less GUS expression when compared to −1210 bp deletion (Figure 5A). Similar pattern was evidenced in stems (Figure 5B); while in roots all the segments respond well as compared to the 35S-GUS construct (Figure 5C). GUS activity was also measured through fluorometric assay and the results are shown in Figure 5D, but due to similar expression pattern of D1 and D2, the data of D2 is omitted in the Figure 5D because of the space constraint. Approx. 5–6.5 fold increase *GUS* expression was observed in roots for full-length, −1210 bp and −519 bp construct compared to control when induced by ABA. While, in leaves and shoots ∼3.5 fold *GUS* induction was observed only for −1210 bp. It is interesting that the full-length, −1210 bp and −886 bp promoter segments positively responded to ABA induction, while no response was detected in leaves and shoots for −519 bp promoter segment under ABA treatment (Figure 5A, B & D). This implied that an ABA-related cis-acting element might exist outside the region of −519 bp construct. We also expose transgenic leaves to SA which failed to induce GUS activities for all constructs (data not showed).

MeJA is a plant hormone involved in tendril (root) coiling, flowering, seed and fruit maturation. An increase in MeJA levels affects flowering time, flower morphology and the number of open flowers. MeJA induces ethylene-forming enzyme activity, which increases the amount of ethylene necessary for fruit maturation. Increased amounts of methyl jasmonate in plant roots have shown to inhibit their growth. Treatments with MeJA for 24 h proved sufficient to trigger *GUS* expression driven by OsPMCa^2+^ATPase promoter constructs (Figure 5). The MeJA induced *GUS* activity level was higher in promoter deletion −1210 bp and −886 bp in case of leaves (Figure 5A). Deletion to −1478 to −1210 bp regions causes rapid increase in *GUS* activity and this might be due to removal of some transcription factors binding regions responsible for MeJA induced gene expression. On the other hand, in shoots, deletion of −886 bp and −519 bp gave higher expression when exposured to MeJA (Figure 5B). Fluorometric data induced by MeJA showed ∼4.3 fold increase GUS activity in leaves for −1210 bp and ∼3.6 fold increases in shoots for −510 bp when compared with control. Approx. 3–4.66 fold increase *GUS* expression was observed in roots for full-length, −1210 bp and −519 bp construct compared to control when induced by MeJA (Figure 5D). The variation of activity in shoot might be due to present of some cis-acting elements outside the deletion −519 bp that causes expression of *GUS* activity in −519 bp and might not present −1478 to −1210 bp regions which causes for the expression in leaves.

### In situ Detection of GUS Activity in OsPMCa^2+^ATPase Promoter Segments Exclusively in Vascular Elements

**Figure 6:**
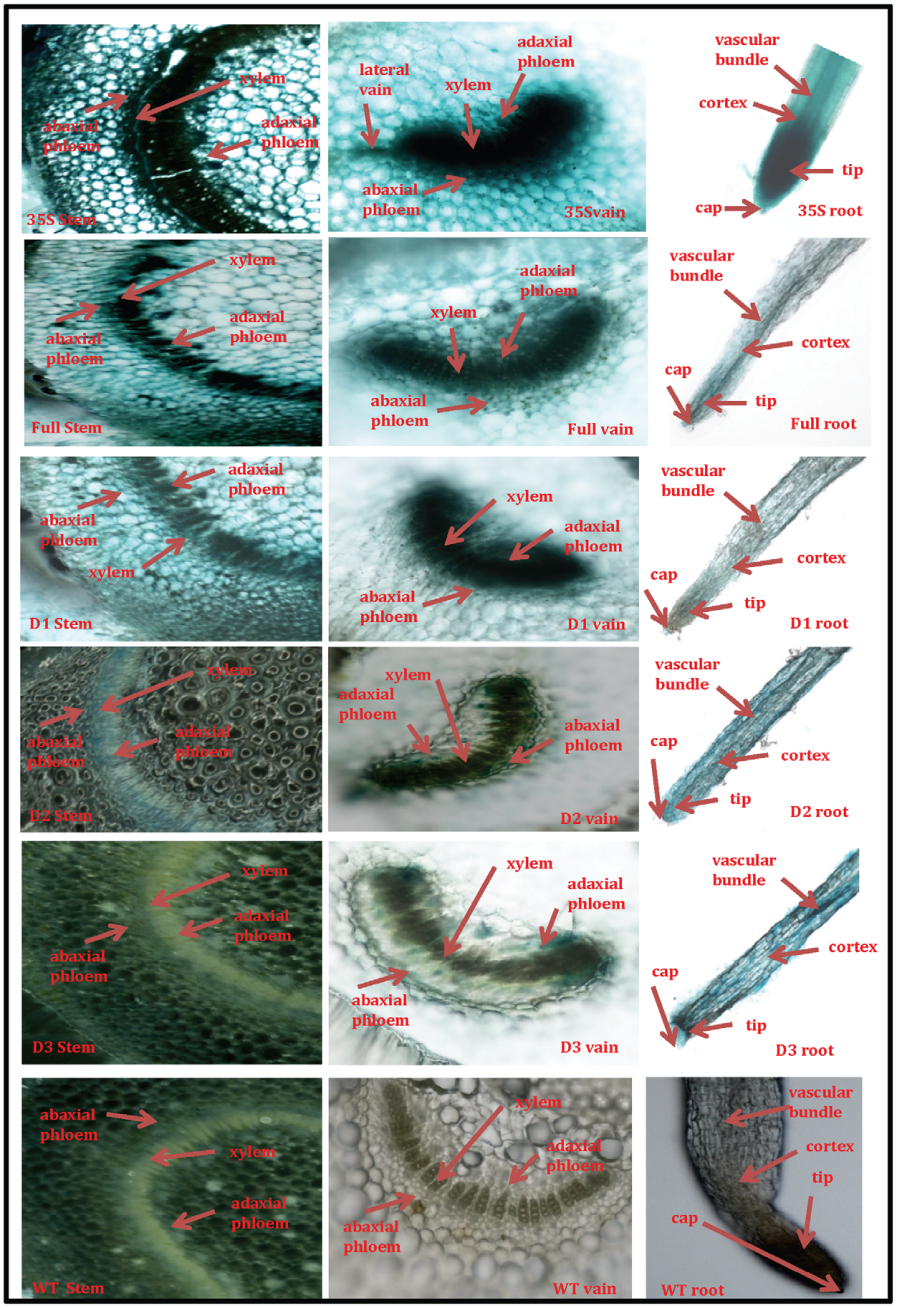
In situ histochemical localization of GUS activity. Razorblade sections in roots, petioles and stems of transgenic tobacco plants transformed with promoter-GUS fusion constructs F, D1 D2 and D3 35S and WT. Transverse petiole and stem sections and from F, D1 D2 and D3 35S and WT were stained with X-Gluc in NaH2 P04, pH 7.0, overnight at 37°C. Root sections from the above constructs were also stained overnight with X-Gluc as above. Positive and negative controls were 35S and WT, used separately.

A *GUS* staining experiment by razorblade section was used for tissue-specific expression of OsPMCa^2+^ATPase promoter, two month-old transgenic containing full-length and its deletions tobacco plants were used. Transgenic plants driven by the CaMV35S promoter, and wild-type tobacco, were the respective positive and negative controls. Blue points were detected in vascular bundles, mesophyll and cortex of leaves; vascular bundles, pith and cortex of stems; and vascular bundles, tips and caps of roots of transgenic tobacco driven by the CaMV35Spromoter (Figure 6). No GUS staining was observed in tissues of wild-type tobacco. A significant induction of GUS expression in the vascular tissue, especially xylem and phloem of leaf veins, stems (Figure 6) was observed in the transverse section of the stems and petioles for transgenic tobacco driven by promoter segment −1478 bp, −1210 bp and −886. While no vascular specific expression was observed in stem section for −519 bp construct but in petiole some expression was observed. In addition, Gus expression was highly detected in the meristematic zone of root tips of transgenic tobacco driven by the OsPMCa^2+^ATPase promoter segment while weakly detected in the root cap. We conjecture that the OsPMCa^2+^ATPase promoter was a vascular specific and particularly a phloem-specific promoter. Moreover, the full-length promoter (−1478 bp) construct still showed the strongest GUS expression activity of the four OsPMCa^2+^ATPase promoter constructs examined.

### GUS Expression Analysis in Reproductive Organs of OsPMCa^2+^ATPase Promoter Segments

**Figure 7:**
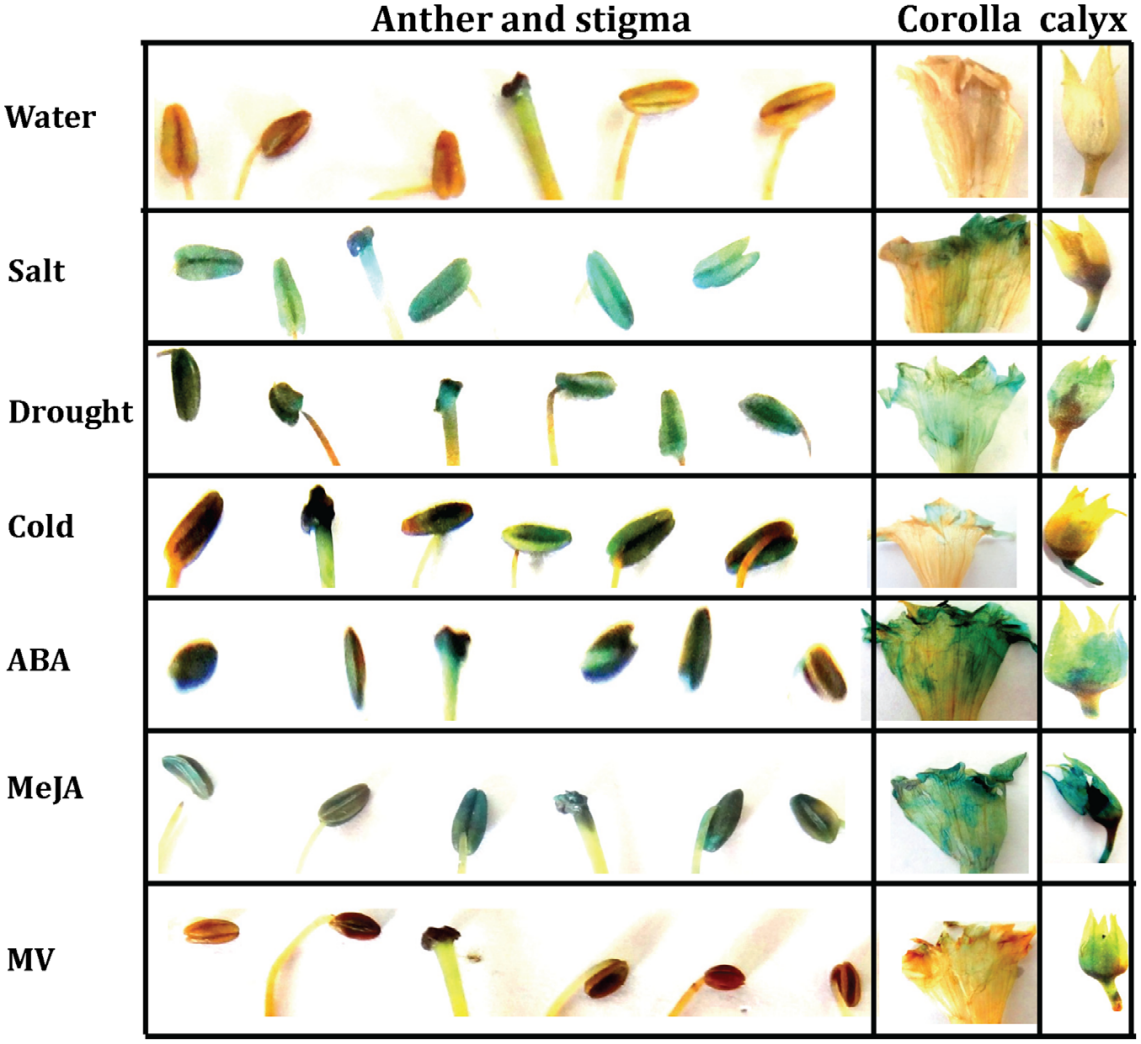
GUS localization and intensity in floral parts of transgenic tobacco plants transformed with OsPMCa^2+^ATPase promoter deletions. Transgenic tobacco flowers, anthers, stigma, corolla and calyx were colored with GUS staining solution to study the localization and intensity under different stress condition.

To investigate the effect of abiotic stresses on the promoter expression in vegetative organs, GUS staining was performed in mature flower following the described stress treatments (Figure 7). Results showed differential expression patterns upon stress treatment. Following salt stress for 24 h, the full-length segment presented high levels of GUS expression in anther and stigma, while relatively low expression was observed in corolla and stalk and no expression in calyx. Drought stress gave similar expression pattern for anther and stigma, however, high expression was evidenced in corolla and calyx and no expression in stalk. Relatively low level of expression was observed for all floral parts upon treatment with cold. Surprisingly, no expression was evidenced in anther and stigma while variable expression levels were evidenced in corolla, calyx and stalks in different deleted fragments (Figure S2). Following MV treatment, no GUS activity was found in anther and stigma when OsPMCa^2+^ATPase full promoter and deletions were used. In some deleted segment low expression was found for corolla, calyx and stalks (Figure 7 & Figure S2). Treatment with ABA and MeJA we observed very high level of GUS expression in anther, stigma as well as other floral organs in case of full-length OsPMCa^2+^ATPase promoter (Figure 7), while differential expression was found for corolla, calyx and stalks in deletion analysis (Figure S2). Some important cis-acting elements present outside the regions of deletion −519 bp could inhibit proper functioning of the deleted segment. This result implies that only full-length OsPMCa^2+^ATPase promoter is sufficient for GUS expression in flower.

## Discussion

Relevant genes can be expressed in most plant tissues either by constitutive promoters such as CaMV35S or inducible-promoters. However, the presence of transgenes driven by constitutive promoters may result in homology-dependent gene silencing, particularly when the promoter is highly active [35]. Inducible-promoters are highly organized sequences required for the correct spatial and temporal gene expression [36]. The advantages of these promoters derived from plant genes make them a potentially powerful tool for improving plant resistance to abiotic and biotic stresses. Hence, tissue-specific and inducible promoters are preferred as experimental tools to analyze the effects of transgene expression and produce transgenic plants with resistance to various abiotic stresses.

The present study evidenced that OsPMCa^2+^ATPase promoter sequence in rice harbored multiple stress cis-acting elements. It has been reported that drought-induced elements usually exist upstream of genes induced by drought stress [37], [38]. Additionally, several transcription binding factors which take part in abiotic stress response can contain cis-elements like ABA responsive element (ABRE), drought responsive elements (DRE), C-repeat elements (CRT), low temperature responsive element (LTRE), or MYC and MYB recognition sites [39]–[41]. Most transcription binding factors are conserved among orthologous, paralogous, and co-regulated genes [42]. Motifs often act in concert with other transcription binding factors in order to cover an effect on abiotic stress response, as in the case of DRE/CRT element. Some transcription binding factors occur within a specific distance from one another, thereby forming dyad elements. This is the case in ABRE element in *Arabidopsis* and *Oryza sativa* [43]. ABRE elements often occur in multiple copies, thereby providing a quantitative effect on stress response. ABRE and DRE elements are also known to occur within different kinds of promoters induced by cold, drought, and salt stress.

Many abiotic stress-responsive motifs exert their effect on the basal transcription machinery, while others lie farther upstream within the promoter. Regulatory elements, in general, form diverse regulatory networks, each having an effect on one another [44]. Within this network, certain transcription factors (TFs) are induced by abiotic stress. They are usually included in DREB, WRKY, MYB, bHLH (basic helix-loop-helix), bZIP, and NAC TF families. Protein-protein interactions between different transcription factors also take part in abiotic stress response [45]. An example is given by the SCOF-1 protein (soybean cold inducible factor-1) which interacts with SGBF-1 (soybean G-box binding bZIP transcription factor) in response to cold stress [46]. Response to abiotic stress can be regulated by ABA-dependent and ABA-independent pathways [47], [48] which overlap each other, and play a major role in response to cold, drought and salinity stress [49]. This result implies that OsPMCa^2+^ATPase promoter could be induced by drought, salt, and cold by activating different signal transduction pathways.

OsPMCa^2+^ATPase expression was induced by pathogen and abiotic elicitors. Several putative motifs within cis-acting elements, such as GAAAAA, TTTTTC, ACGT-box and W-box, were evidenced by computational analysis in the OsPMCa^2+^ATPase promoter and they might be responsible for OsPMCa^2+^ATPase expression during pathogen infection and salt stress. An −1210 bp sequence of OsPMCa^2+^ATPase promoter was sufficient to drive GUS activity in tobacco leaf, shoot, root and flower challenged with NaCl stress. Cis-acting elements essential for activation in response to salt may reside between −1210 and −519 bp. Only a GT-1 element identified in the soybean calmodulin gene promoter activated by pathogen infection and NaCl stress was found within the OsPMCa^2+^ATPase promoter region from −1210 to −519 bp [50]. The presence of the GT-1 element suggests that it may function in OsPMCa^2+^ATPase promoter activation in response to bacterial infection and salt stress. GA in the GT-1 cis-element (5′-GAAAAA-3′) is required for binding to nuclear factor(s) in response to pathogen or salt-induced stress [50]. We also find GT-1-related elements in OsPMCa^2+^ATPase promoter which imply that a GT-1-related transcription factor positively regulates OsPMCa^2+^ATPase gene expression under the conditions of pathogen attack or NaCl stress. In our result we also identified some GT-1 element at −519 to −1 bp but under salt stress these segments did not respond, indicating that this GT-1 element may not be sufficient for OsPMCa^2+^ATPase promoter activation by salt treatment. Only −1210 bp promoter segments responded in leaves and shoots of tobacco transgenic plants submitted to MV treatments. Oxidative damage in plants caused by MV may be due to the excess generation of superoxide radicals, which are normally detoxified to oxygen and hydrogen peroxide (H_2_O_2_) by superoxide dismutase [51].

The plant growth regulators appear to play a predominant role in the conversion of environmental signals into changes in plant gene expression [52] and are involved in diverse developmental processes including root growth, pollen production, and plant resistance to insects and pathogens [53], [54]. The ABA-responsive, bZIP transcription factor-binding ACGT-box, and EREs were found in the OsPMCa^2+^ATPase promoter region. The −519 bp did not respond to ABA treatment in case of leaves and shoots as we found no cis-regulatory element in this region. The −593 bp deletion construct did not respond to ABA treatment [55], although ABA responsive bZIP and MYB binding sites were found in this region. The GCC-box-like jasmonic acid-responsive element was evidenced in the OsPMCa^2+^ATPase promoter region. The −1210 to −886 bp is sufficient for MeJA-induced GUS activity in OsPMCa^2+^ATPase promoter. However, the −519 bp deletion drastically reduced jasmonic acid-responsive promoter activity in leaves but not in shoots and roots. This may be due to the fact that some inhibitory elements might be present in this region. Previous studies have implicated a number of different types of regulatory elements in conferring MeJA-responsiveness in plant promoters. Some shares sequences containing the TGACG (or its inverse CGTCA) motif, as in the case of the AS-1-type element in the glutathione S-transferase gene [56] or JASE1 and JASE2 elements in OPR1 (12-0xo-phytodienoic acid-10, 11-reductase) gene [57]. The palindromic motif CGTCA - TGACG is part of longer inverted repeats in case of lipoxygenase 1 (*Lox l*) gene [58], the potato cathepsin D inhibitor gene [59] and nopaline synthase (nos) gene [60]. Such TGACG-containing elements have been previously identified as binding sites for bZIP-type of transactivating factors [61]. We found a TTGAC element within the OsPMCa^2+^ATPase promoter region located at −1261 bp. It had been suggested to bind with SA-dependent and pathogen-induced transcription factors WRKY and TGA [62], [63]. However, in our case, the promoter constructs containing these cis-acting elements were not activated by SA treatment.

Transverse section of the stems and petioles indicated that the OsPMCa^2+^ATPase promoter was a phloem-specific promoter. It is interesting that in some deleted fragment GUS activity did not occur in xylem but only in phloem, so long as the cambium was formed. Moreover, root sections showed that staining was not only in the vascular bundle but also in the root tip meristematic zone. It was also interesting that GUS staining was weekly detected in root cap, which differed to results for the CaMV35S promoter. The mechanism behind this is still unclear. The members of the DOF TF family have been found to control vascular tissue-specific gene expression by binding to the core recognition sequence CTTT [64]. Consistent with this, eight similar motifs were also found in the OsPMCa^2+^ATPase promoter. Consequently, this confirmed that the motif ‘CTTT’ and its homologous sequences determined the promoters’ phloem-specific expression pattern. Other conserved motif like ATAAGAACGAATC also involved in the phloem strength and specificity was identified by Hehn [65]. Other vascular-specific promoters from rice, Milk Vetch Dwarf Virus [66] and pumpkin PP2 gene promoter [67] also contained this conserved sequence. In all these promoters, this motif was upstream of the TATA box [48]. Our results demonstrated that OsPMCa^2+^ATPase promoter has role in survival adaptability by responding to stresses and hormones.

To attain anther-specific transgene expression, an anther specific promoter is necessary. Full-length OsPMCa^2+^ATPase promoter showed very high level of GUS expression in anther, stigma as well as other floral organs upon treatment with NaCl, drought, ABA and MeJA, while relatively low expression was evidenced in case of cold-induced stress. No anther and stigma specific activity was observed under MV treatment. Surprisingly, none of the deleted fragments showed any expression for anther and stigma, while varied expression was found in corolla, calyx and stalks. This might be due to the presence of repressor elements located outside the deleted fragment, which could block the activity in anther and stigma. This is the first report where OsPMCa^2+^ATPase promoter conferred anther-specific transgene expression under various abiotic and hormonal stresses. The results presented here suggest that the OsPMCa^2+^ATPase promoter may play an important signalling and/or defensive role during the flowering stages. Previously it was reported that MeJA induced flower specific expression in Tomato Prosystemin Promoter [68]. Similar flower-specific expression patterns have been detected in several other MeJA-responsive genes in tomato [69], [70], soybean [71] and tobacco [72], [73], whereas stigma-specific genes that also respond to MeJA have been detected in *Arabidopsis* [74], [75], and rice [76]. Interestingly, signalling and stress related genes are frequently present in stigma and most of them also share several cis-regulatory elements. In contrast with dicot plants, anther-specific promoters are not well characterized in monocot plants. As such, no cis-acting elements for anther-specific expression have been identified in rice. Although several anther-specific promoters have been isolated from monocots, their specificity was evaluated only in dicot transgenic plants [77]–[79]. For instance, rice OSIPA and OSIPK promoter regions share two cis-elements (GTGANTG10 and POLLEN1LELAT) that confer anther-specific expression to a minimal promoter in dicots [79]. However, the functionality of these cis-elements is still unknown.

Plasma membrane proteins are involved in the recognition and transduction of endogenous hormonal signals [80]. However, SA had no effect on OsPMCa^2+^ATPase promoter expression in tobacco leaves. Induction of disease resistance-related plasma membrane proteins by plant hormones has not been reported. Cold-regulated plasma membrane protein genes are induced in wheat and rice by ABA treatment [81]–[83]. Inducible OsPMCa^2+^ATPase promoter may be efficient at mediating and enhancing plant defense responses against abiotic stresses.

### Conclusion

In the present study we demonstrated by histochemical analysis that the full-length OsPMCa^2+^ATPase promoter from *Oryza sativa* is a reproductive organ-specific as well as vascular-specific promoter. The OsPMCa^2+^ATPase promoter contains drought, salt, cold, MV, ABA and MeJA related cis-elements, which regulated gene expression in roots. It was also revealed that in the leaves these cis-elements might exist outside the region contained in the −519 bp deletion construct. Overall, the tissue-specificity and inducible nature of OsPMCa^2+^ATPase promoter could grant wide applicability in plant biotechnology. Abiotic stress tolerance in rice is a complex trait, and single transgene introduction may not be sufficient to impart stress tolerance under field conditions. Further strategies involving rice genetic transformation with multiple transgenes expressed in an inducible manner will help to improve its stress tolerance. In consequence, the promoter analyzed in the present study could be of great use to drive transgenes based on expression pattern and extent of required inducibility.

## Supporting Information

Figure S1 **PCR analysis to detect the presence of different promoter deletions in transgenic tobacco. A)** PCR of full-length promoter **B)** PCR of D1 promoter deletion **C)** PCR of D2 promoter deletion and **D)** PCR of D3 promoter deletion.
(PDF)

Figure S2 **GUS localization and intensity in floral parts of transgenic transformed with different deleted promoter segments.** **A)** GUS localization and intensity in tobacco flowers/florets for D1. **B)** GUS localization and intensity in tobacco flowers/florets for D2. **C)** Reproductive organs GUS localization and intensity in tobacco flowers/florets for D3. Flower parts were stained with GUS staining solution to study the localization and intensity under different stress condition. Details of stress treatments are described in material and methods.
(PDF)

Table S1 **List of primers used in this study.**
(PDF)
