# Supplementary material for: Reproductive Organ and Vascular Specific Promoter of the Rice Plasma Membrane Ca2+ATPase Mediates Environmental Stress Responses in Plants
Source: PLoS One. 2013 Mar 1;8(3):e57803. doi: 10.1371/journal.pone.0057803 (PMC3585799; doi:10.1371/journal.pone.0057803)
Supplement: Figure S1 — PCR analysis to detect the presence of different promoter deletions in transgenic tobacco. A) PCR of full-length promoter B) PCR of D1 promoter deletion C) PCR of D2 promoter deletion and D) PCR of D3 promoter deletion. (PDF) [file pone.0057803.s001.pdf]

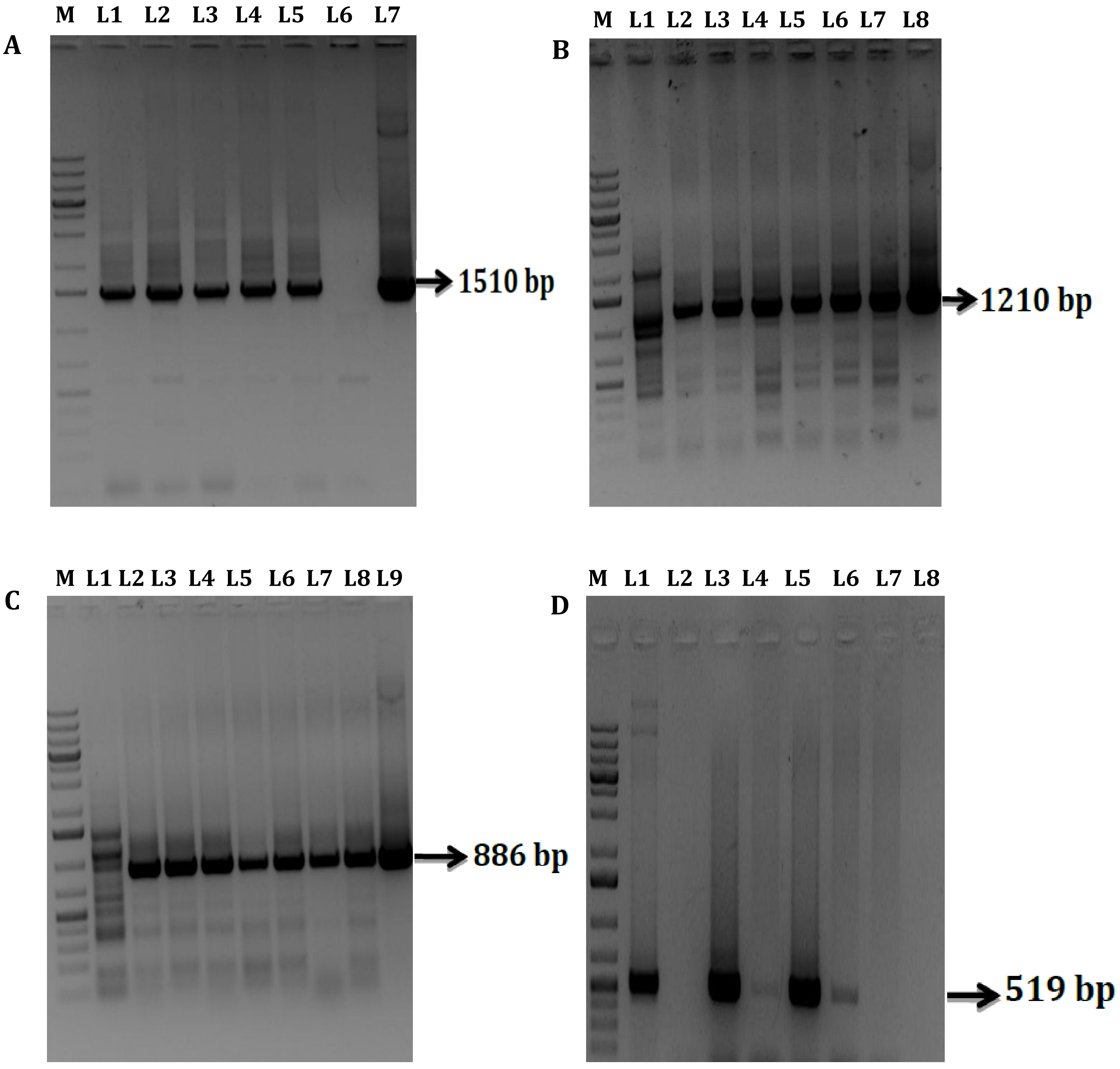

Figure S1: PCR analysis to detect the presence of different promoter deletions in transgenic tobacco. **A)** PCR of full length promoter **B)** PCR of D1 promoter deletion **C)** PCR of D2 promoter deletion **D)** PCR of D3 promoter deletion.
