# Supplementary material for: Reproductive Organ and Vascular Specific Promoter of the Rice Plasma Membrane Ca2+ATPase Mediates Environmental Stress Responses in Plants
Source: PLoS One. 2013 Mar 1;8(3):e57803. doi: 10.1371/journal.pone.0057803 (PMC3585799; doi:10.1371/journal.pone.0057803)
Supplement: Figure S2 — GUS localization and intensity in floral parts of transgenic transformed with different deleted promoter segments. A) GUS localization and intensity in tobacco flowers/florets for D1. B) GUS localization and intensity in tobacco flowers/florets for D2. C) Reproductive organs GUS localization and intensity in tobacco flowers/florets for D3. Flower parts were stained with GUS staining solution to study the localization and intensity under different stress condition. Details of stress treatments are described in material and methods. (PDF) [file pone.0057803.s002.pdf]

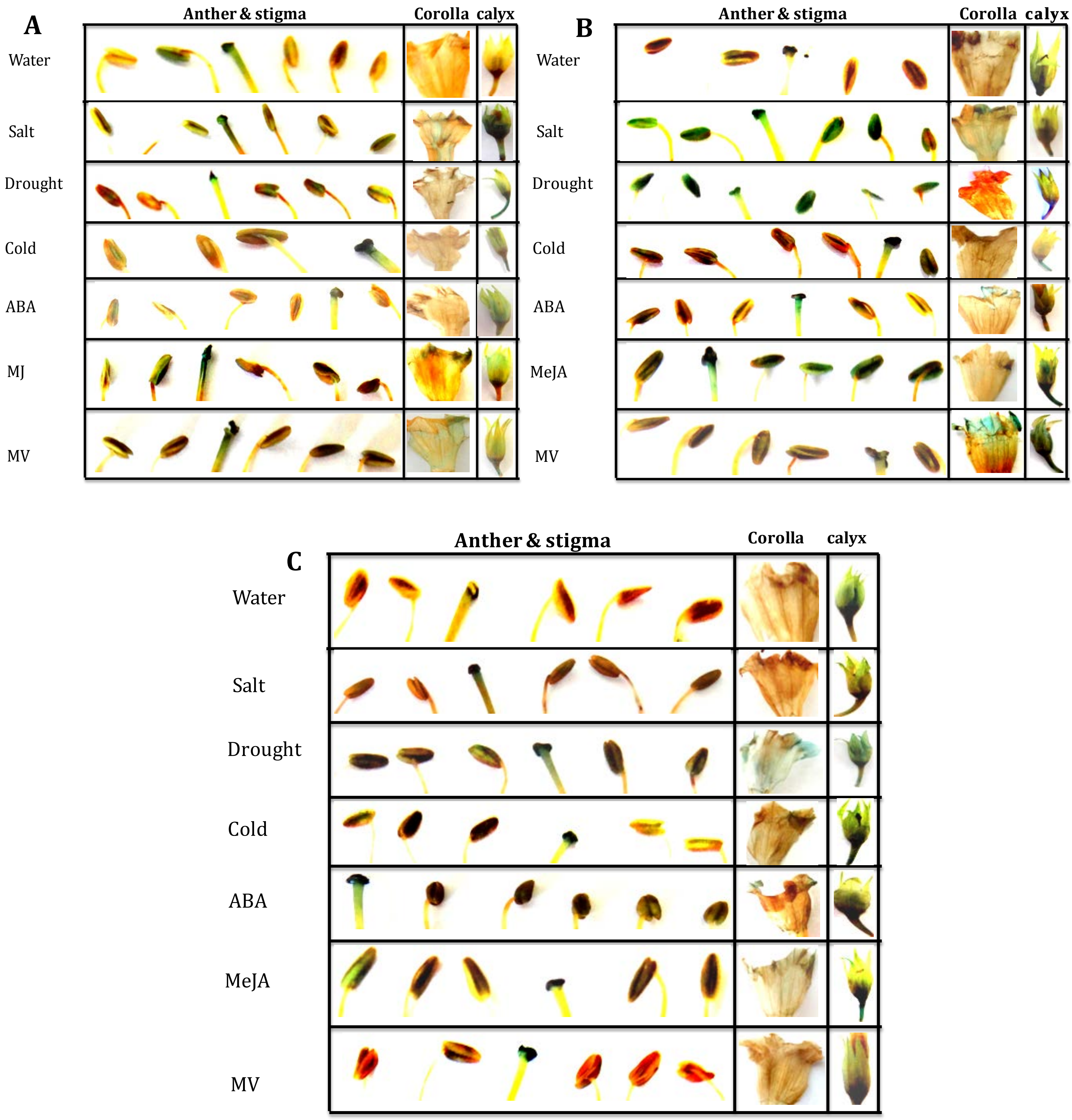

Figure S2: GUS localization and intensity in floral parts of transgenics transformed with different deleted promoter segments. **A)** GUS localization and intensity in tobacco flowers/florets for D1 **B)** GUS localization and intensity in tobacco flowers/florets for D2 **C)** Reproductive organs GUS localization and intensity in tobacco flowers/florets for D3. Flower parts were stained with GUS staining solution to study the localization and intensity under different stress condition. Details of stress treatments are described in material and methods.
