# Supplementary material for: Reproductive Organ and Vascular Specific Promoter of the Rice Plasma Membrane Ca2+ATPase Mediates Environmental Stress Responses in Plants
Source: PLoS One. 2013 Mar 1;8(3):e57803. doi: 10.1371/journal.pone.0057803 (PMC3585799; doi:10.1371/journal.pone.0057803)
Supplement: Table S1 — List of primers used in this study. (PDF) [file pone.0057803.s003.pdf]

Table S1. List of primers used in this study

Primers Name	Primers Sequence
Full pro FP	5’ CAAGCTTCGTGCTTGCATGTCACTTTTTATG 3’
D1 FP	5’ CAAGCTTTGATATTCACTCCTGTAGTTAA 3’
D2 FP	5’ CAAGCTTCGTCCACTTTATGCCAAAAGA3’
D3 FP	5’ CAAGCTTCGCAATAGACAACTCGAAGTCATATCG 3’
Full pro RP	5’ CGGGATCCCTGGTCGCTAGTGAAGACGA 3’
HYG-FP	5’ GGTTTCCACTATCGGCGAGT 3’
HYG-RP	5’ GCGACGTCTGTCGAGAAGTT 3’
GUS FP	5’ GAGGCT AAT TCG GCT ATG ACT G 3’
GUS RP	5’ ATCGGG AGA GGC GAT ACC GTA 3’
